# CrAssphage distribution analysis in an Amazonian river based on metagenomic sequencing data and georeferencing

**DOI:** 10.1128/aem.01470-24

**Published:** 2025-04-25

**Authors:** David Tavares Martins, Oscar Victor Cardenas Alegria, Carlos Willian Dias Dantas, Edian Franklin Franco De Los Santos, Paulo Rógenes Monteiro Pontes, Rosane Barbosa Lopes Cavalcante, Rommel Thiago Jucá Ramos

**Affiliations:** 1 Laboratory of Bioinformatics and Genomics of Microorganisms, Federal University of Pará-UFPA 37871, Belém, Pará, Brazil; 2 Institute of Biological Sciences, Federal University of Pará-UFPA, Belem, Pará, Brazil; 3 Laboratory of Simulation and Computational Biology — SIMBIC, Federal University of Pará 37871, Belém, Pará, Brazil; 4 Center of High Performance Computer and Artificial Intelligence — CCAD, Federal University of Pará, Belem, Pará, Brazil; 5 Institute of Biological Sciences, Federal University of Minas Gerais 113014, Belo Horizonte, Minas Gerais, Brazil; 6 Campus Central de Herrera, Universidad Tecnológica de Santiago 370455, Santo Domingo, Dominican Republic; 7 Vale Institute of Technology - Sustainable Development, Belém, Brazil; Universidad de los Andes, Bogotá, Colombia

**Keywords:** CrAssphage, biomarker, fecal contamination, Amazon, freshwater

## Abstract

Viruses are the most abundant biological entities in all ecosystems of the world. Their ubiquity makes them suitable candidates for indicating fecal contamination in rivers. Recently, a group of Bacteroidetes bacteriophages named CrAssphages, which are highly abundant, sensitive, and specific to human feces, were studied as potential viral biomarkers for human fecal pollution in water bodies. In this study, we evaluated the presence, diversity, and abundance of viruses with a focus on crAssphages via metagenomic analysis in an Amazonian river and conducted correlation analyses on the basis of physicochemical and georeferencing data. Several significant differences in viral alpha diversity indexes were observed among the sample points, suggesting an accumulation of viral organisms in the river mouth, whereas beta diversity analysis revealed a significant divergence between replicates of the most downstream point (IT4) when compared to the rest of the samples, possibly due to increased human impact at this point. In terms of the presence of crAssphage, the analysis identified 61 crAssphage contigs distributed along the Itacaiúnas River. Moreover, our analysis revealed significant correlations between 19 crAssphage contigs and human population density, substantiating the use of these viruses as possible markers for human fecal pollution in the Itacaiúnas River. This study is the first to assess the presence of crAssphages in an Amazonian river, with results suggesting the potential use of these viruses as markers for human fecal pollution in the Amazon.

**IMPORTANCE:**

The Amazon biome is one of the most diverse ecosystems in the world and contains the most vast river network; however, the continuous advance of urban centers toward aquatic bodies exacerbates the discharge of pollutants into these water bodies. Fecal contamination contributes significantly to water pollution, and the application of an improved fecal indicator is essential for evaluating water quality. In this study, we evaluated the presence, diversity, and abundance of crAssphages in an Amazonian river and performed correlation analysis on the basis of physicochemical and georeferencing data to test whether crAssphages are viable fecal pollution markers. Our analysis revealed both the presence of crAssphages and their correlation with physicochemical data and showed significant correlations between the relative abundance of crAssphages and human density. These results suggest the potential use of these viruses as markers for water quality assessment in Amazonian rivers.

## INTRODUCTION

The Amazon biome is one of Earth’s most expansive and ecologically diverse ecosystems (1). It contains a vast river network discharging approximately 18% of the available global freshwater to the Atlantic Ocean (2). Nevertheless, due to the relentless advance of human activities toward the forest periphery, the proximity of urban centers to aquatic bodies is increasing, exacerbating the discharge of pollutants and enabling the potential dissemination of pathogens within these ecosystems (3–5).

The most widely used method for examining water quality is the detection of fecal indicator bacteria (FIB), which are indicators of water contamination, with the bacteria including fecal coliforms, *Escherichia coli*, *Streptococcus*, and *Enterococcus* (6, 7). However, the exclusive use of bacteria for fecal contamination detection poses limitations since FIB are present in both human and animal feces, making it impossible to detect contamination sources (8). FIB correlates poorly with human viral pathogens (9), which are relatively persistent in water environments and resistant to current wastewater treatment methods (10). Additionally, monitoring all viral pathogens is time-consuming and cost-prohibitive for most developing countries, so there is an urgent need for a universal marker that can be easily implemented in microbiological routines (8).

Viruses are ubiquitous organisms that are distributed throughout all ecosystems around the globe, with their abundance estimated to be nearly 10^31^ virus-like particles (11–13). They are obligate parasites in many organisms, and the most prevalent group consists of bacteriophages, which are viruses that infect archaea and bacteria (14). Dutilh et al. (15) first described the most abundant bacteriophages present in the human gut, crAssphages. Since their discovery, this group of bacteriophages that infect bacteria from the phylum *Bacteroidetes* (16) has been evaluated as a possible biomarker for human fecal contamination (17). They have promising characteristics, such as high abundance in sewage water, high human feces specificity, high correlation with human viral enteric pathogens, and low replication rates outside the human gut (8, 18–20). The use of these viruses as fecal biomarkers may circumvent the limitations of current bacterial indicators (21); thus, it is necessary to evaluate the presence of these viruses in regions such as the Amazon.

We used metagenomic shotgun analysis to explore the abundance and diversity of crAssphages in the Itacaiúnas River in Marabá, Pará, Brazil. We compared the results with the physicochemical parameters used in routine water quality analysis and with human population density and deforestation data to correlate the river’s degree of anthropization with crAssphage abundance. To our knowledge, this is the first study to analyze crAssphage presence in an Amazonian river and to correlate it with georeferencing data to determine biomarkers to be used for environmental health surveillance.

## MATERIALS AND METHODS

### Sampling area

The city of Marabá (PA), Brazil, was chosen as the focal area, as it lies along the course of the Itacaiúnas River, a significant waterway within the Itacaiúnas River Basin with an area of 42,000 km². It encompasses 10 municipalities in the southeastern region of the state of Pará, including Marabá, Curionópolis, Eldorado dos Carajás, Parauapebas, São Geraldo do Araguaia, Canaã dos Carajás, Piçarra, Água Azul do Norte, Xinguara, and Sapucaia. The Itacaiúnas River is the primary conduit within this basin, spanning over 390 km (22).

The water samples were collected in triplicate from four distinct sampling locations along the course of the Itacaiúnas River: IT1 (5°24′09″S, 49°08′00″W), IT2 (5°21′57″S, 49°05′36″W), IT3 (5°21′24″S, 49°07′19″W) and IT4 (5°21′18″S, 49°08′33″W). In total, 12 water samples were collected for analysis. The Itacaiúnas River traverses the city of Marabá, Pará, which spans an expansive area of 15,128.058 km² and is inhabited by a population of 266,536 individuals, as per the data from the Brazilian Institute of Geography and Statistics (23). This urban center plays a pivotal role as a regional hub and is particularly noteworthy for its prominence in the domain of mineral extraction, with a primary focus on resources such as copper, manganese, and iron (23).

### Sample collection and measurement of physicochemical parameters

The 12 freshwater samples were collected (from four points in triplicate) via a Van-Dorne bottle, which was decontaminated with 10% hypochlorite and distilled water prior to the collection of each replicate. The acquired samples were subsequently transferred into 5-L polypropylene containers for each of the replicates. A total of four physicochemical variables were measured via a HANNA HI 9828 multiparameter instrument: pH; electrical conductivity (EC) (μS/cm); total dissolved solids (TDS) (ppm); and water-dissolved oxygen content (ppm).

### Sample preprocessing and DNA extraction

The collected samples were preprocessed via two filtration steps: initially, the samples were subjected to negative pressure filtration through a 14-µm nitrocellulose membrane (Qually) to facilitate the removal of large solid particles. Next, the filtered samples were processed via a 0.22-µm nitrocellulose membrane (Millipore) to retain biological materials. The nitrocellulose membranes, which included the biological components, were subsequently transferred to polypropylene 50-mL Falcon tubes containing a DNA-preserving solution composed of 50-mM Tris–HCl, 500-mM NaCl, and 125-mM EDTA (pH 8.0). These prepared samples were then stored at a temperature of −80°C to maintain the integrity of the genetic material prior to subsequent metagenomic DNA extraction procedures.

DNA extraction was carried out with a DNeasy PowerSoil Pro Kit (QIAGEN) in accordance with the manufacturer’s recommended protocols. To assess the concentration and purity of the extracted DNA, a NanoDrop 1000 spectrophotometer was used. The integrity of the DNA was further evaluated via gel electrophoresis, with 0.5-µg/mL ethidium bromide incorporated during the electrophoresis process. Samples with DNA concentrations equal to or greater than 50-ng/µL and with a purity index within the range of 1.4–2.0 were selected for subsequent sequencing analyses.

### Georeferencing analysis

The methodology for mapping the anthropic signature of land use and land cover in rivers is based on the concept of a hydrographical basin and geoprocessing techniques. The deforestation level and population density were evaluated following the methodology of Cavalcante et al. (24). The deforested area was calculated as the sum of all areas with nonnatural land use and land cover based on the classification of the Mapbiomas Brazil project (https://brasil.mapbiomas.org/en/) version 6 for the year 2020. The sampling points were chosen following a bioprospecting approach, with consideration given to the gradient of anthropogenic impact and accessibility for sample collection and are listed in the previous section.

### Sequencing and bioinformatics

#### Sequencing library and quality control

The extracted DNA was sequenced via the Illumina NovaSeq 6000 platform with a 2 × 150-bp paired-end library. Next, the quality of the sequenced reads was evaluated with the FastQC platform v0.11.2 (25), and the low-quality reads were removed with Fastp v0.22.0 (26) using default parameters.

#### CrAssphage identification

The reads from each replicate were merged by sample and assembled with Megahit v1.2.9 (27). Furthermore, the contigs were provided to VirSorter2 v2.2.4 (28) (parameters: keep-original-seq; include-groups dsDNAphage, ssDNA, NCLDV, Lavidaviridae; min-length 5,000; min-score 0.5) and DeepVirFinder v1.0 (29) (parameters -l 500) for viral sequence prediction. Then, contigs with scores ≥ 0.95 were submitted to CheckV v1.0.3 (checkv-db-v1.5) (30) to check for viral genome completeness. On the basis of the protocol of Guo et al. (31), the contigs were maintained if their length was ≥3 kb, viral gene ≥1, if contig length ≥5 kb, viral genes = 0, and host genes = 0, or if contig length ≥10 kb, viral genes = 0, and host genes = 1. Contigs were removed if their length was ≥50 kb, if there were more than 3× more cellular-like genes than viral-like genes, and if no proviral signals were assigned by CheckV. Moreover, the contigs were annotated via the DRAMv pipeline v1.5.0 (32), and a final filtration step was performed. Contigs with zero DRAMv viral hits were removed, and on the basis of the list of potentially suspicious genes provided by Guo et al. (31), contigs with >50% annotations containing suspicious genes were removed. Finally, the filtered contigs were input into the Integrated Phage HOst Prediction pipeline v1.3.3 (IPHOP - database: iPHoP_db_Aug23_rw) (33) for host prediction and were also input into geNomad v1.11.0 (34) for contig taxonomic assignment. The contigs assigned to the *Crassvirales* order were extracted and used for posterior analysis. Finally, the completeness of the filtered contigs was again evaluated via CheckV (30).

#### Statistical analysis

All contigs were used to construct a reference index via Bowtie 2 v2.4.2 (35). Then, the reads were mapped to the contigs, and the read mapping data were retrieved via SAMtools v1.12 (36). The relative abundances of the contigs were calculated by normalizing the reads via the transcripts per million method (TPM), and the relative abundances were log (x + 1) transformed. The contigs predicted to be viral were extracted from the read mapping tables generated by Bowtie 2 and SAMTools.

The relative abundances of the viral contigs were used to calculate alpha and beta diversity indices via the Vegan package (37). For alpha diversity, the Shannon and species richness indices were used, and differences were calculated with an analysis of variance test in *R*. For beta diversity, Bray‒Curtis distances and a permutational multivariate analysis of variance (PERMANOVA) test were performed to check for differences in data dispersion with the adonis2 package (38). Moreover, a cladogram was plotted via Ward’s hierarchical agglomerative clustering method (39). All of the utilized statistical tests were performed in R.

The contigs classified as *Crassvirales* by geNomad were extracted, and then, the normalized abundances were used to plot a cluster map with the seaborn Python library (40). The seaborn cluster map function calculates the Euclidean distances between rows and columns and then performs hierarchical clustering on the data via agglomerative clustering. Finally, the correlations between crAssphages relative abundances and environmental parameters were calculated. The normality of the data was evaluated via the Shapiro‒Wilk test, and the Spearman correlation coefficient was calculated. Statistical tests were performed via the Python SciPy library (41).

## RESULTS AND DISCUSSION

### Sequencing and taxonomy overview

Metagenomic sequencing yielded approximately 409.3 million reads at the four sampling points, with three replicates each (12 samples), with an average of 34.1 million reads per replicate. The quality assessment results revealed an average Phred quality score of 35 across all the reads, which were free from adapter contamination (Table S1).

### Viral composition and diversity

**Fig 1:**
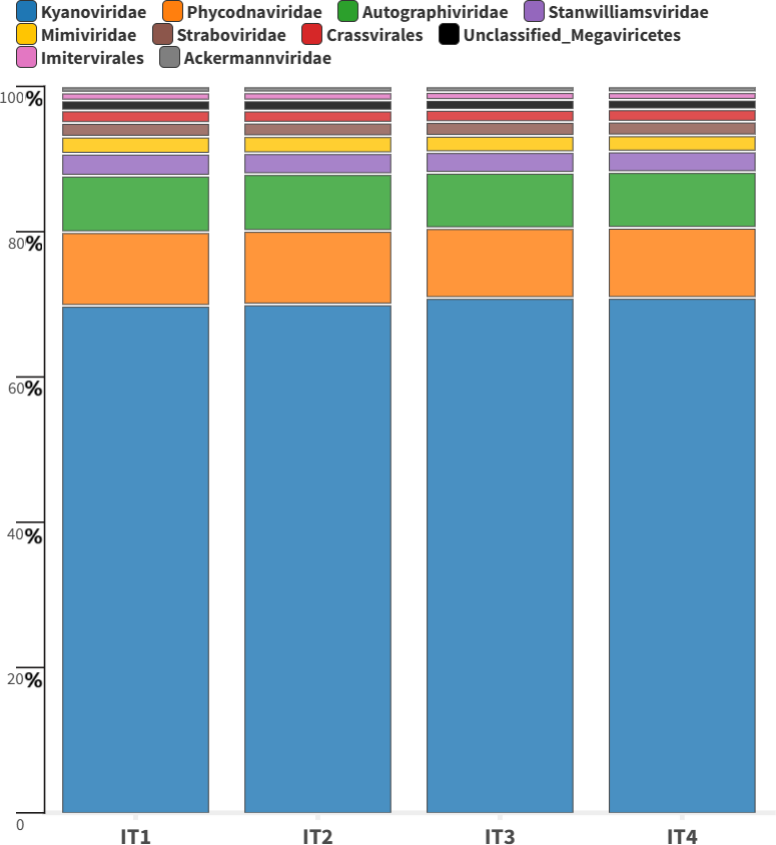
Viral contigs classified by geNomad. Phages classified in the *Crassvirales* order are represented in red.

A contig-based approach was taken to retrieve putative viral genomes. The viral sequence prediction procedure was able to extract 18,210 viral contigs, which were further taxonomically classified by geNomad into 20 viral families and three orders (Fig. 1). The majority of the reads mapped back to the viral contigs were classified only at the order taxonomic level in the *Caudoviricetes* order; 80.41% and 1.3% did not receive any classification by geNomad.

Regarding the classified viral data, at the family level, the Itacaiúnas virome was dominated by cyanophages, primarily from the family Kyanoviridae, constituting 70.38% of the log(TPM + 1) transformed reads. Furthermore, Phycodnaviridae algae viruses (9.87%), Autographiviridae bacteriophages (7.72%), Stanwilliamsviridae (2.89%), Mimiviridae protist viruses (2.29%), and Straboviridae (1.88%) were detected. These results align with other freshwater virome studies performed in river ecosystems, in which a greater prevalence of cyanophages than bacteriophages and eukaryotic viruses was detected (3, 42, 43). On the basis of this viral ecosystem, geNomad was able to identify 61 contigs belonging to bacteriophages from the *Crassvirales* order, which represented 1.69% of the virome data.

**Fig 2:**
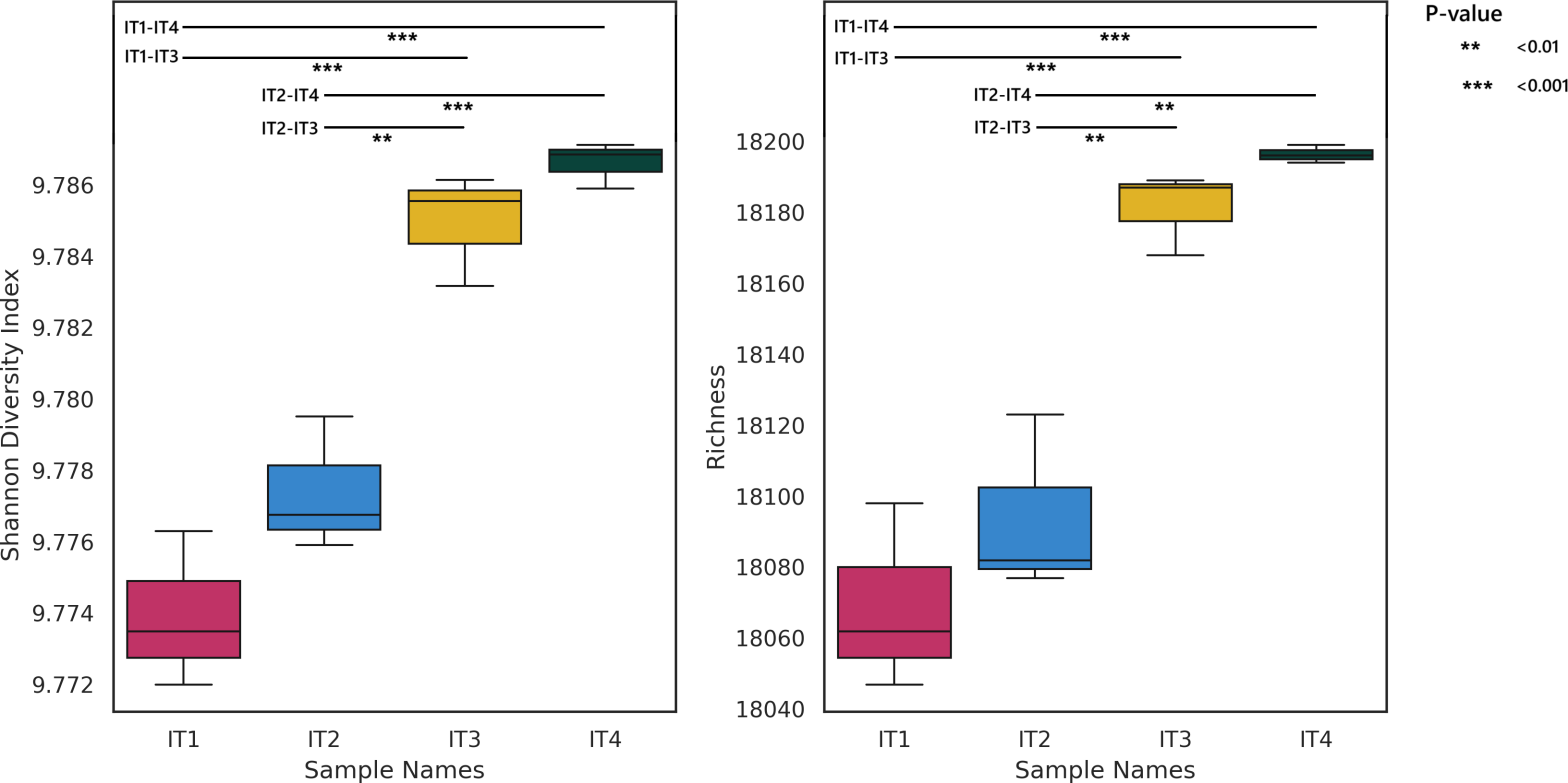
Shannon index and richness of the viral population across the Itacaiúnas River classified on the basis of the relative abundances of viral contigs as identified by geNomad along with *P* values of post hoc Bonferroni tests. In the *y*-axis scales, significant differences between the viral diversity of the sample points are highlighted.

In terms of viral diversity, the raw reads mapped to the viral contigs were used to calculate alpha and beta diversity indices. For alpha diversity, the Shannon and richness indices revealed a significant increase in viral diversity along the course of the Itacaiúnas River. Both indices showed significant differences between the means of samples (Shannon index: *F* = 39.31; *P* < 0.001; richness index: *F* = 32.62; *P* < 0.001). In terms of Shannon index, both the IT1 and the IT2 samples significantly differed from IT3 and IT4 (Fig. 2). A similar pattern was observed for the richness index (Fig. 2).

Previous studies have shown that land use, local geography, weather events, and tidal effects may affect how viral particles are deposited and accumulate in different parts of rivers (43, 44). The alpha diversity results in the current study suggest that human activities at the margins of the Itacaiúnas River may generate an accumulation of viral organisms in the river mouth since points IT3 and IT4 are closest to the most populated areas of Marabá city.

**Fig 3:**
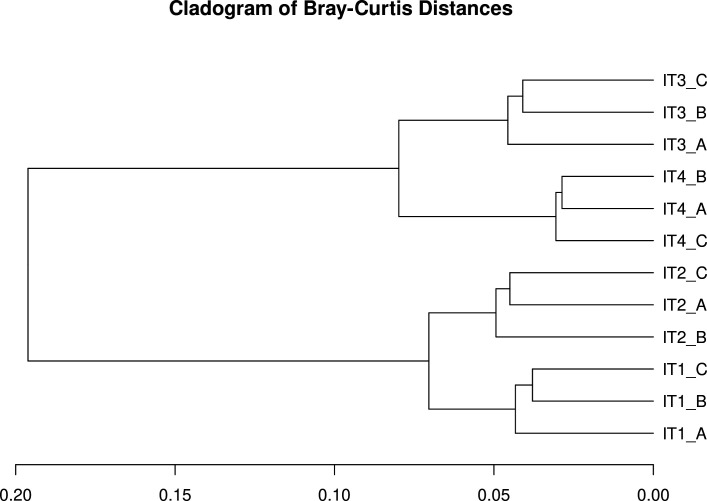
Viral beta diversity represented with Bray‒Curtis distances in a dendrogram. The samples are labeled according to collection site.

For beta diversity analysis, the relative abundances of the viral contigs were used to calculate Bray‒Curtis distances, which were then clustered via the Ward algorithm and plotted in a dendrogram (Fig. 3). It is possible to observe the presence of four major clusters of samples, with replicates of samples IT1 and IT2 close to each other and those of samples IT3 and IT4 also close.

PERMANOVA utilizing the Bray‒Curtis index revealed significant differences among the four sampling points (*F* value: 5.09; *P* value: 0.003). Pairwise PERMANOVA revealed a significant dissimilarity between the IT4 samples and the remaining samples (Table S3). The dissimilarity of most downstream points in the Itacaiúnas River might represent differences in levels of anthropogenic impact since the IT4 sampling point is the putatively most anthropogenically impacted point and is the point surrounded by the highest human density; in contrast, the other sampling points are near areas with lower population densities.

### CrAssphage presence and abundance

Contigs classified in the *Crassvirales* order were filtered, and their relative abundances were used in further methodological steps. The relative abundance of crAssphages subtly increased from IT1 to IT2, followed by progressive increases at points IT3 and IT4 (Fig. S1). The metagenomic data together with the georeferencing data suggest that there are potential sources of fecal contamination upstream of point IT1 in the city of Marabá, and within the city’s territory, contamination becomes more apparent after point IT2.

**Fig 4:**
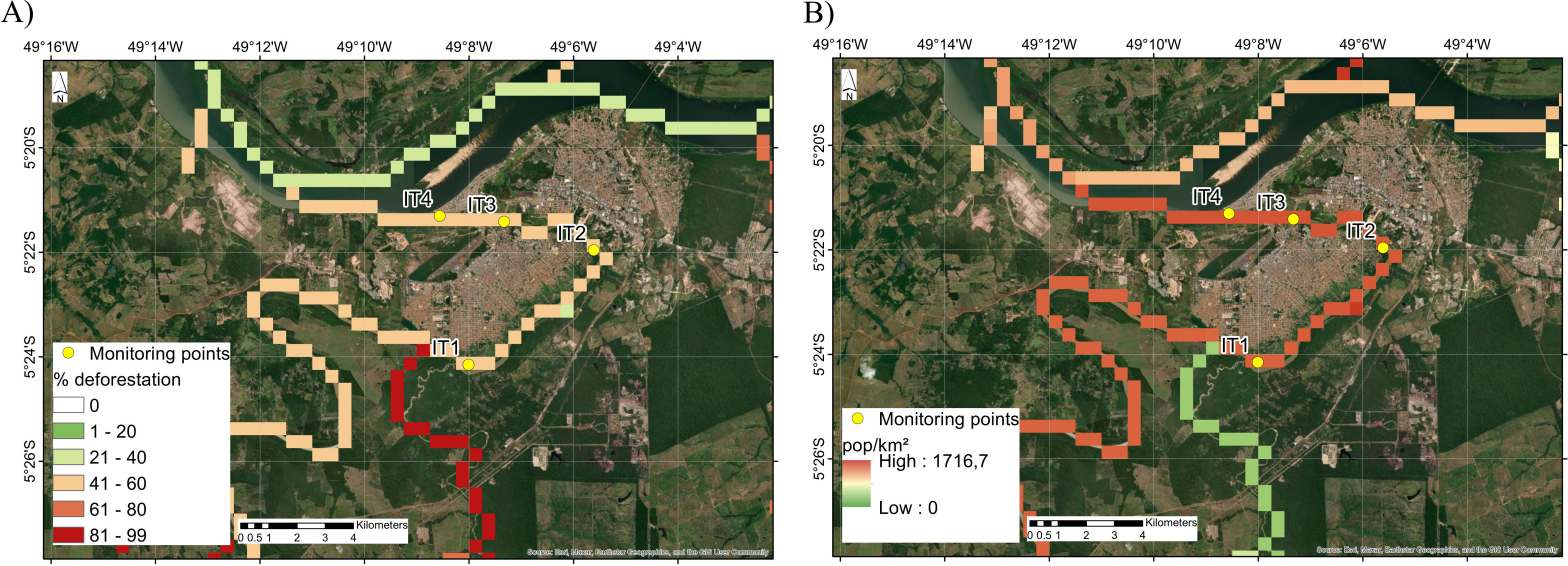
(**A**) Percentage of deforestation in areas adjacent to the sampling points. The intensity is represented by color, with red representing high, yellow indicating medium, and green showing low levels of deforestation. (**B**) Population density by km^2^ in areas adjacent to the sampling points. The intensities of the values are represented by the same colors used in panel A. The maps were generated by using ArcMap 10.8 together with a Fortran in-house script.

A potential contamination source at point IT1 could be a tributary river of the Itacaiúnas River, which is surrounded by areas with high levels of deforestation (53.51%), as depicted in Fig. 4. Although the population levels in the region are not high, the high levels of deforestation may be indicative of human settlement. Furthermore, human expansion toward forests is typically associated with the settlement of communities with limited urbanization and poor sanitation, which could contribute to the discharge of fecal waste into water (1).

CrAssphages were identified at all sampling points, and this might be useful for identifying areas with existing fecal contamination in the Itacaiúnas River and for initiating surveillance to prevent public health problems. This is consistent with previous studies in which crAssphages were detected in recreational waters in Toronto (45) and in putatively pristine sites from South African rivers (46). This information can be used for further decision-making by the government to intervene in problems related to water quality. Notably, the IT1 and IT2 sampling points are close to recreational areas, and the presence of crAssphages in these areas may represent a risk for human health since crAssphages are highly associated with human gastrointestinal viral pathogens. For example, Jennings et al. (19) detected crAssphage covariation with norovirus in Chile, and Crank et al. (47) detected crAssphage correlation with human polyomavirus in Italian wastewater. Given that bacteria may have a synergistic relationship with viruses pathogenic to humans, paving the way for viral infection (48) and even increasing viral infectivity (49), the action of crAssphages in the human gastrointestinal tract microbial community could also mediate the bacteria‒virus relationship, explaining the existing correlation between these pathogenic viruses and crAssphages.

### CrAssphage contig distribution

**Fig 5:**
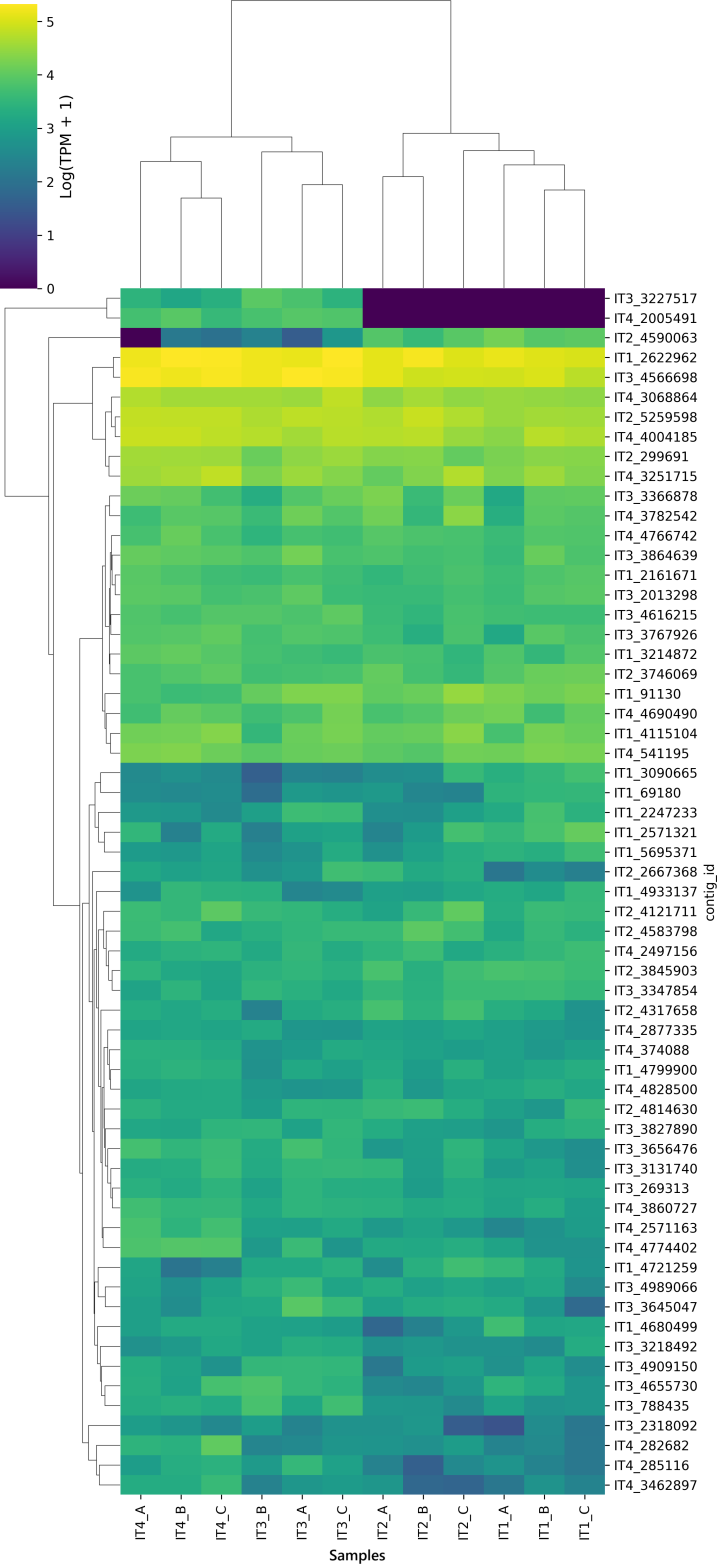
The relative abundances of crAssphage contigs as identified by geNomad. The relative abundances were calculated by mapping reads to the contigs that were normalized via TPM and transformed to log10(TPM + 1). The *x*-axis labels represent the sample names, while the *y*-axis labels represent the contig names.

To assess differences in crAssphage contig distribution along the course of the Itacaiúnas River, agglomerative clustering of the relative abundance of the contigs was performed, and the results were plotted in a cluster map (Fig. 5). The analysis revealed a clear separation between samples IT1 and IT2 and between samples IT3 and IT4, indicating that the crAssphage profile in more anthropized environments differed from that in less human-impacted environments. Two of the 61 contigs showed an exclusivity pattern since they were present only in samples IT3 and IT4 and were completely absent in samples IT1 and IT2 (IT3_3227517 and IT4_2005491). This observation aligns with findings by Mafumo et al. (46), who noted crAss-like contigs present exclusively in human-impacted sites but absent in less impacted environments; however, in the present research, we could not guarantee that points IT1 and IT2 were free from human interference.

### Characterization and host prediction of crAssphage contigs

The completeness assessment for crAss-like contigs as evaluated by CheckV indicated that 61 contigs presented a low completeness score (< 50% complete). An *in silico* exploration of crAssphages within the Itacaiúnas river virome resulted in the recovery of 14 contigs from IT1, 10 from IT2, 19 from IT3, and 30 from IT4.

**Fig 6:**
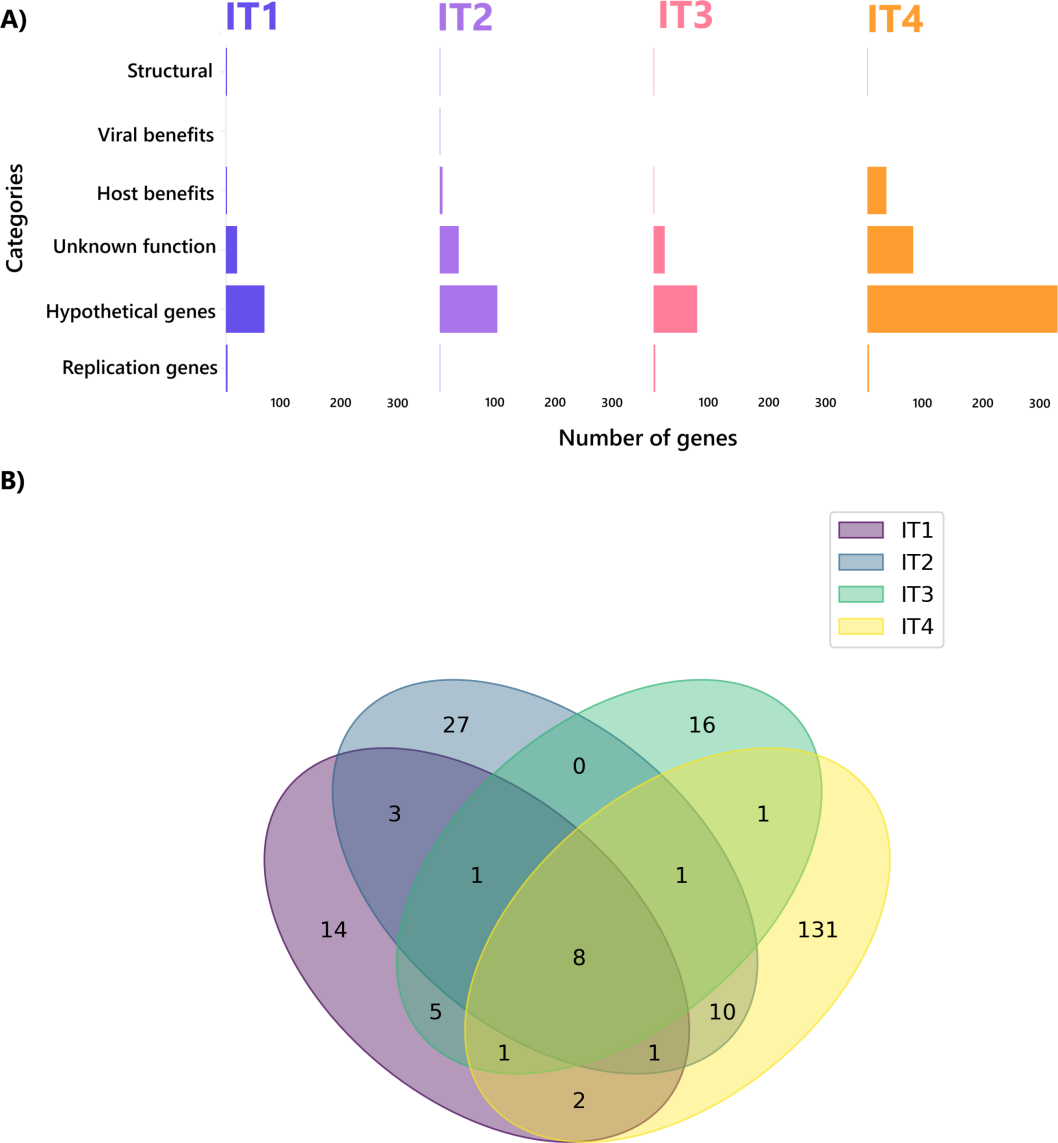
(**A**) CrAssphage gene categories at the four sampling points. The genes are divided into six different categories. Viral structural genes: genes responsible for the physical structure of viruses; viral genes with viral benefits: genes that encode proteins that increase the virus’s ability to infect, replicate, and disseminate throughout the host; viral genes with host benefits: genes that can modulate host cellular processes in a way that benefits the host; viral genes with unknown function: genes with unknown functions but with experimentally validated existence; viral genes with hypothetical functions: genes that are predicted *in silico* but are not experimentally validated; viral replication genes: genes that encode proteins that are directly involved in the replication of the viral genome. (**B**) Distribution of unique crAssphage genes along the sample points.

With respect to crAssphage contig annotation via the DRAMv software, the analysis revealed 804 genes categorized into six categories: hypothetical viral genes (73.38%) (viral genes detected *in silico* but not validated), viral genes with unknown functions (19.4%) (viral genes that are experimentally validated but whose function is unknown), viral genes with host benefits (5,22%), viral genes with viral benefits (0.12%), viral replication genes (1.24%), and viral structural genes (0.62%). In terms of crAssphage marker genes, the analysis identified eight genes that were present at all sample points (Fig. 6). Among these genes, we detected the terminase large subunit and portal protein, two of the three conserved capsid and genome-packaging proteins of crAssphages (TerL, portal, and MCP) (50), which are used as hallmark genes. Some other interesting genes included HAD family hydrolase, pyrophosphatase, and DNA polymerase (Table S4). These genes could be identified as possible candidate marker genes for crAssphage detection in the Itacaiúnas River, but further studies are needed to confirm their specificity for crAssphage identification.

Moreover, a host prediction analysis revealed putative hosts for four crAssphage contigs retrieved from the sample points IT2, IT3, and IT4. Among these putative hosts, two were classified in the *Bacteroida* phylum, one was classified as *Cyanobacteriota*, and one wasas classified as an *Archaea*. This association with *Cyanobacteriota* and *Archaea* has never been reported by a study and may possibly be caused by IPHOP database limitations. Another possibility is that since environmental *Crassvirales* phages share the same environment as archaeal and cyanobacterial viruses, they may have some level of genetic material exchange which may confound host taxonomy predictors. It is also necessary to state that only fragments of crAssphages were retrieved in these samples, which limits the precision of phage host prediction tools.

In early studies, crAssphages were thought to infect only bacteria from *Bacteroida*, and efforts to cultivate them in *Bacteroides intestinalis* (51) and *Bacteroides thetaiotaomicron* (52) were ultimately successful in the isolation of crAssphages. However, recent studies based on *in silico* prediction have revealed crAssphages that potentially infect bacteria in the phyla *Pseudomonadota*, *Bacillota*, and *Verrucomicrobiota* in both the human microbiome and in environmental samples (46, 53).

As previously reported by Mafumo et al. (46), crAssphage may associate with other hosts depending on environmental niche. Although it is early to state that *Crassvirales* phages may infect cyanobacteria or archaea (54), in Mafumo et al. (46), the knowledge on crAssphage diversity continues to grow, and new studies are continually reporting that crAssphages replicate in environments other than the human gut microbiome; these environments include the gut microbiomes of mammals such as dogs, cats, cattle, and pigs (18, 55, 56) and even the environment (57). Understanding crAssphage diversity is essential for differentiating which crAssphages exclusively infect bacteria from the human gut and which species can possibly replicate in the environment, facilitating the identification of new crAssphage markers.

### Influence of environmental factors on crAssphage distribution

The physicochemical parameters measured at the Itacaiúnas River are detailed in Table S2. Given the variations in viral communities across different sampling points, efforts were made to comprehend how biological factors influence viral distribution in the river. To this end, correlation tests were employed to evaluate the biological factors potentially influencing crAssphage differences. First, only correlations between environmental factors (physicochemical and georeferencing data) were determined, and then, correlations between the relative abundances of crAssphage contigs and environmental parameters were assessed.

In Brazil, environmental management laws are governed by the National Environmental Council (CONAMA), which is the Brazilian agency that provides the National Environmental Policy related to the National Environmental System. The CONAMA Resolution 357/2005 pertains to environmental guidelines for the classification of water bodies on the basis of measurements of water physicochemical and biological parameters.

With respect to the suitability of the parameters by the CONAMA Resolution, dissolved oxygen (DO) is crucial in sustaining aquatic life because it impacts biological and biochemical processes in water bodies; high DO values aid in rapid pollutant degradation, whereas low DO levels impede this process (58, 59). The CONAMA Resolution 357/2005 stipulates DO levels above 5 ppm for river water, and all points evaluated in this study recorded values above the minimum limit, with IT2 showing the lowest value: 6.22. EC is a measure used to detect how much electrical current a solution can conduct, whereas TDS represents the total weight of dissolved mineral elements per unit volume of water (60). CONAMA sets the TDS limit at 500 mg/L and does not describe either the EC or the EC standard. According to the California State Water Resources Control Board, the EC standard values for good freshwater range from 100 to 2,000 μS/cm. In the Itacaiúnas River, EC and TDS values remained within the stipulated specifications at all the sampling points, and both were significantly correlated with each other (Spearman’s *r*: 0.93/*P* value: 1.1 × 10^−5^) and were positively correlated with DO EC vs. DO: Spearman’s *r* = 0.7/*P* value <0.05; TDS vs. DO: Spearman’s *r* = 0.79/*P* value <0.05) (Fig. S2). Since EC, TDS, and DO may serve as indirect indicators of pollution (60, 61), their correlation is consistent with the literature data.

**Fig 7:**
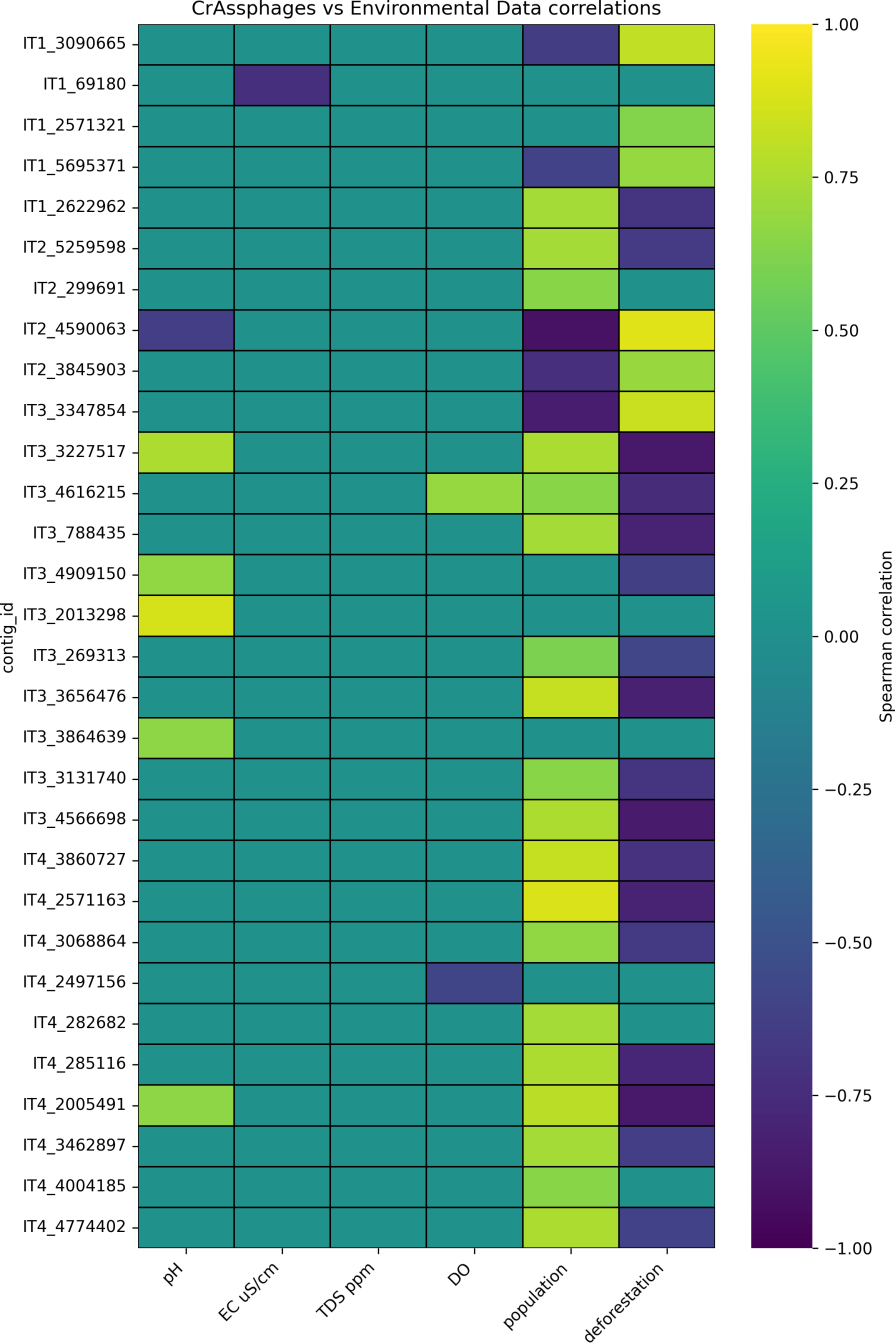
Correlations of crAssphage contig relative abundance with environmental parameters. The purple values indicate negative correlations, whereas the yellow values indicate positive correlations. The *x*-axis labels represent the sample names while the y-axis labels represent the contig names.

In terms of the correlation of the relative abundance of crAssphages with environmental data, we identified a series of significant Spearman’s correlations (Fig. 7), but important considerations exist. Overall, no strong correlation was observed between crAssphages and the physicochemical parameters, suggesting that these factors do not explain the observed variation in crAssphage abundance. Given that pH, DO, EC, and TDS are known to influence phage stability, decay, and persistence in the environment (62–64), some degree of correlation with crAssphage abundance would have been expected. The most likely explanation for this lack of significant correlations is the limited variation in physicochemical parameters, as all measured values in the Itacaiúnas River complied with the CONAMA Resolution standards. Specifically, pH values, which should range between 6 and 9 to support aquatic life and human consumption, remained within this acceptable range. Consequently, the measured crAssphage correlations are based on parameters that do not necessarily indicate risks to human consumption. These results indicate that physicochemical parameters alone may not be enough to determine human pollution in rivers, as the analysis revealed a considerable number of crAssphage contigs at river sites with parameters suitable for aquatic life maintenance.

Considering that crAssphages are supported as markers of human fecal pollution, the discussion will focus primarily on the correlation of crAssphages with human density. The analysis revealed that 19 crAssphage contigs were positively correlated with the population, which substantiates their use as potential markers for human fecal pollution. Correlation between several crAssphage contigs and human density was not detected, since many *Crassvirales* phages can be found naturally in the environment (57). Nevertheless, some of the uncorrelated crAssphage contigs may still work as feasible markers for the detection of human fecal pollution because river flow can lead to pollution in areas that are far from the origin points of pollution, as described in the “CrAssphage presence and abundance” section. Interestingly, a substantial number of crAssphage contigs (five contigs) were negatively correlated with population density. One possible cause for this arises from the location at which these contigs were retrieved (IT1 and IT2). Although these sampling points are supposed to contain a small population and thus have lesser anthropogenic impact than points IT3 and IT4, these locations are normally used as recreational sites, which have a higher human concentration during weekends than during weekdays. This means that even in putatively less impacted environments, the presence of crAssphages indicates the dissemination of human fecal pollution along the Itacaiúnas River course.

Nevertheless, it is necessary to consider that the majority of studies concerning crAssphage presence and abundance in water bodies consider only specific quantitative PCR (qPCR) markers that do not account for the true diversity of crAssphages in environmental samples. Since the metagenomic approach allows for the visualization of a larger picture of crAssphage composition, it is possible to identify new crAssphage species that are not necessarily related to human feces.

From the 19 human-correlated crAssphage contigs, an evaluation of gene content was performed. We identified a set of four genes that are both present in the crAssphages and found in all of the locations that include a crAssphage portal protein, a TerL protein, a crAssphage DNA polymerase, and a hypothetical protein KNV36_gp052. These genes need to be further evaluated to test their taxonomic resolution for identifying crAssphages and could be used in the future for the development of qPCR or digital PCR assays for the fast and inexpensive detection of crAssphages in Amazonian freshwater bodies.

### Limitations

The main limitations of the current study are related to the sampling locations. Unfortunately, it was not possible to collect water from points further upstream and downstream of the city of Marabá, which restricted the analysis to sampling points only within the city limits. Furthermore, none of the water qualities of the samples were considered inappropriate according to the CONAMA Resolution, which limits our ability to correlate crAssphage with physicochemical pollution indicators. Furthermore, despite the existence of substantial data in the literature on the correlation between crAssphages and human viral enteric pathogens, we did not perform concentration measurements of viral enteric pathogens throughout Itacaiúnas to validate their relationship with crAssphages. Finally, the filtration membrane used, which had a size of 0.22 µm, allowed many viruses to pass through. Although it is necessary to account for viral associations in medium-sized particles, a recent study revealed that crAssphages may be significantly associated with particles in the size range of 0.45–0.2 µm (65), which would be retrieved by a 0.22-µm membrane.

### Conclusions

The findings of this study contribute to our current knowledge regarding the distribution of crAssphages in water bodies worldwide. These viruses can be used as markers that accurately detect human influence on the environment through molecular biology and bioinformatic approaches. The analysis resulted in the identification of 61 crAssphage contigs distributed along the course of the Itacaiúnas River, indicating possible unseen dissemination of fecal contamination in the river. The correlation analysis revealed that 19 crAssphages were positively correlated with human population density across the Itacaiúnas River, corroborating their use as possible biomarkers for fecal contamination detection. The current data provide pertinent information regarding the use of crAssphages as fecal pollution biomarkers that, in future studies, could be implemented in qPCR or digital PCR panels for fecal contamination surveillance in Amazonian rivers as well as for decision-making regarding freshwater monitoring and treatment, given their sensitivity and specificity.

## Data Availability

The data presented in the study were deposited in the National Center for Biotechnology Information (NCBI) database under BioProject ID PRJNA1122411. The scripts used for the generation of images can be found in github.com/labgm/CrAssphage_Analysis_Amazon_River_Martins_et_al.

## SUPPLEMENTAL MATERIAL

Supplemental material aem.01470-24-s0001.docx Tables S1 to S4 and Figures S1 and S2.

ASM does not own the copyrights to Supplemental Material that may be linked to, or accessed through, an article. The authors have granted ASM a non-exclusive, world-wide license to publish the Supplemental Material files. Please contact the corresponding author directly for reuse.
