# Supplementary material for: CrAssphage distribution analysis in an Amazonian river based on metagenomic sequencing data and georeferencing
Source: Appl Environ Microbiol. 2025 Apr 25;91(5):e01470-24. doi: 10.1128/aem.01470-24 (PMC12093941; doi:10.1128/aem.01470-24)
Supplement: Supplemental material — Tables S1 to S4 and Figures S1 and S2. [file aem.01470-24-s0001.docx]

Table S1: Quality filtering results

| **Sample** | **Total reads** | **Reads passed quality filters** | **Reads with low quality** | **Reads with too many N** | **Reads too short** | **Reads mapped to viral contigs** |
| --- | --- | --- | --- | --- | --- | --- |
| IT1_A | 74560210 | 73810090 | 717118 | 7312 | 25690 | 2503703 |
| IT1_B | 75436092 | 74818492 | 589748 | 7406 | 20446 | 2992478 |
| IT1_C | 74001388 | 73305070 | 660556 | 7690 | 28072 | 2989581 |
| IT2_A | 72355746 | 71619620 | 699774 | 7528 | 28824 | 2317404 |
| IT2_B | 67060528 | 66392024 | 628954 | 6868 | 32682 | 1807123 |
| IT2_C | 62274932 | 61671942 | 567658 | 6384 | 28948 | 1923590 |
| IT3_A | 60186754 | 59595816 | 556366 | 6260 | 28312 | 2023194 |
| IT3_B | 65123244 | 64391004 | 700140 | 6708 | 25392 | 2448457 |
| IT3_C | 61596106 | 61033076 | 533056 | 6332 | 23642 | 2438772 |
| IT4_A | 67507898 | 66921186 | 554680 | 6838 | 25194 | 3333395 |
| IT4_B | 78869912 | 78124622 | 694462 | 8012 | 42816 | 3928091 |
| IT4_C | 59747138 | 59300428 | 414522 | 6194 | 25994 | 2963776 |

Table S2: Physicochemical parameters, population density and deforestations levels collected from the Itacaiúnas river sample points

|  | | | | | | |
| --- | --- | --- | --- | --- | --- | --- |
| **Sample** | **pH** | **EC uS/cm** | **TDS ppm** | **DO** | **population** | **deforestation** |
| IT1_A | 6,59 | 87 | 61 | 6,41 | 13.02 | 53.51 |
| IT1_B | 6,96 | 87 | 61 | 6,39 | 13.02 | 53.51 |
| IT1_C | 7,05 | 87 | 61 | 6,47 | 13.02 | 53.51 |
| IT2_A | 6,84 | 87 | 61 | 6,34 | 14.44 | 53.47 |
| IT2_B | 6,83 | 88 | 61 | 6,22 | 14.44 | 53.47 |
| IT2_C | 6,85 | 89 | 62 | 6,82 | 14.44 | 53.47 |
| IT3_A | 7,49 | 87 | 61 | 6,66 | 15.83 | 53.46 |
| IT3_B | 7,35 | 91 | 63 | 6,74 | 15.83 | 53.46 |
| IT3_C | 6,98 | 89 | 62 | 6,76 | 15.83 | 53.46 |
| IT4_A | 7,52 | 49 | 34 | 6,37 | 16.06 | 53.46 |
| IT4_B | 6,99 | 89 | 62 | 6,7 | 16.06 | 53.46 |
| IT4_C | 6,9 | 88 | 61 | 6,51 | 16.06 | 53.46 |

Table S3: Pairwise PERMANOVA results

|  | | | | |
| --- | --- | --- | --- | --- |
|  | **IT1** | **IT2** | **IT3** | **IT4** |
| **IT1** | - | 0.153 | 0.492 | 0.025 |
| **IT2** | 0.148 | - | 0.324 | 0.004 |
| **IT3** | 0.502 | 0.328 | - | 0.007 |
| **IT4** | 0.021 | 0.003 | 0.007 | - |
| **PERMANOVA results** F = 5.09 p = 0.003 | | | | |

Table S4: Shared crassphage genes among all sample points

|  |
| --- |
| **viral_hit** |
| YP_010108877.1 HAD family hydrolase [Flavobacterium phage vB_FspP_elemoA_7-9A] |
| YP_010111146.1 hypothetical protein KNV36_gp052 [uncultured phage cr108_1] |
| YP_008240654.1 portal protein [Cellulophaga phage phi4:1] |
| YP_008241505.1 hypothetical protein Phi17:2_gp010 [Cellulophaga phage phi17:2] |
| YP_008242242.2 DNA polymerase [Cellulophaga phage phi14:2] |
| YP_008242272.1 pyrophosphatase [Cellulophaga phage phi14:2] |
| YP_010110248.1 terminase large subunit [uncultured phage cr55_1] |
| YP_010108828.1 calcineurin-like phosphoesterase [Flavobacterium phage vB_FspP_elemoA_7-9A] |


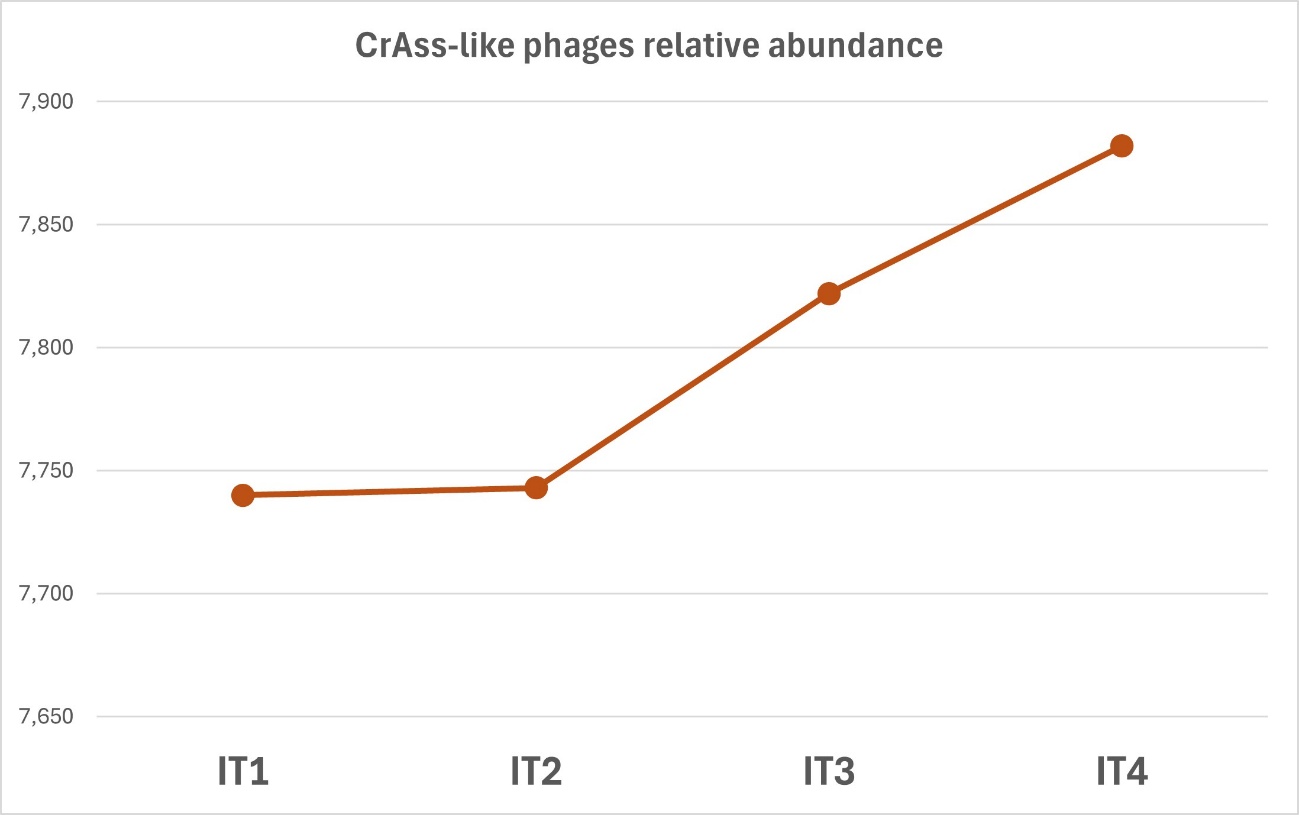


**FIG S1** Total *Crassvirales* phages abundance among the Itacaiúnas river samples


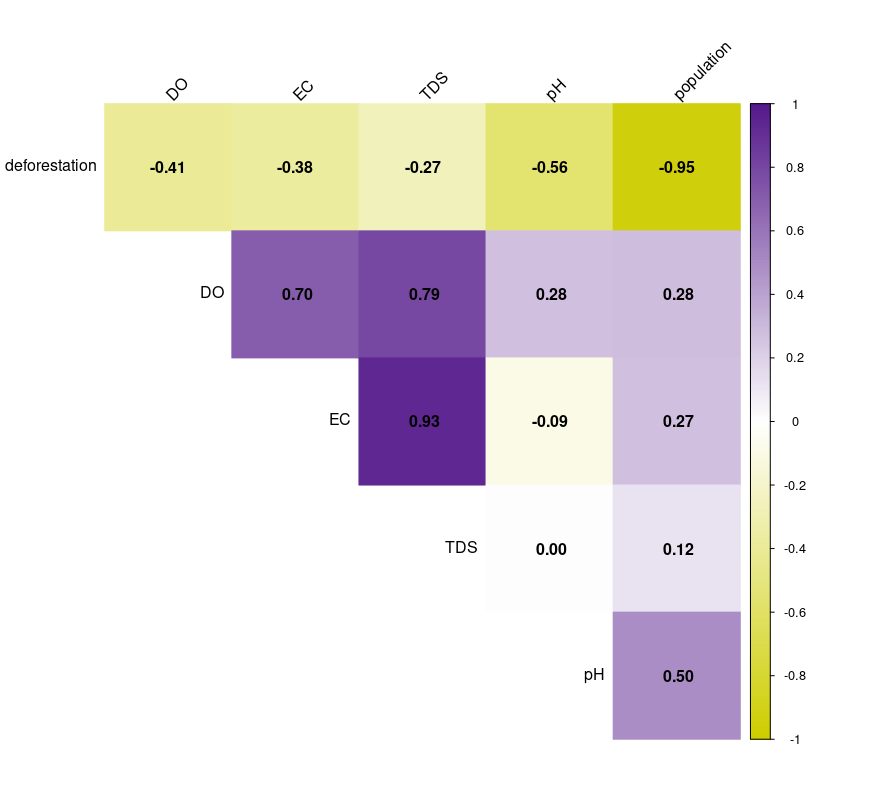
**FIG S2** Evaluated correlations between the environmental parameters
